# Convolutional Neural Networks for Raw Signal Classification in CNC Turning Process Monitoring

**DOI:** 10.3390/s24051390

**Published:** 2024-02-21

**Authors:** Emmanuel Stathatos, Evangelos Tzimas, Panorios Benardos, George-Christopher Vosniakos

**Affiliations:** Manufacturing Technology Laboratory, School of Mechanical Engineering, National Technical University of Athens, Heroon Polytechniou 9, GR15772 Athens, Greece; mstatha@mail.ntua.gr (E.S.); v_tzimas@mail.ntua.gr (E.T.); vosniak@central.ntua.gr (G.-C.V.)

**Keywords:** industry 4.0, smart manufacturing, process monitoring, signal processing, deep learning, part quality prediction, CNC machining

## Abstract

This study addresses the need for advanced machine learning-based process monitoring in smart manufacturing. A methodology is developed for near-real-time part quality prediction based on process-related data obtained from a CNC turning center. Instead of the manual feature extraction methods typically employed in signal processing, a novel one-dimensional convolutional architecture allows the trained model to autonomously extract pertinent features directly from the raw signals. Several signal channels are utilized, including vibrations, motor speeds, and motor torques. Three quality indicators—average roughness, peak-to-valley roughness, and diameter deviation—are monitored using a single model, resulting in a compact and efficient classifier. Training data are obtained via a small number of experiments designed to induce variability in the quality metrics by varying feed, cutting speed, and depth of cut. A sliding window technique augments the dataset and allows the model to seamlessly operate over the entire process. This is further facilitated by the model’s ability to distinguish between cutting and non-cutting phases. The base model is evaluated via k-fold cross validation and achieves average F1 scores above 0.97 for all outputs. Consistent performance is exhibited by additional instances trained under various combinations of design parameters, validating the robustness of the proposed methodology.

## 1. Introduction

Industry 4.0 is characterized by the integration of digital technologies, including Big Data, the Internet of Things, Machine Learning (ML), and cyber-physical systems into manufacturing [1,2]. Cyber-physical systems often appear in the form of Digital Twins, which typically incorporate advanced ML models for monitoring individual machines [3,4] or broader systems [5,6]. These models carry out real-time inference based on data collected from the physical assets, in order to evaluate the monitored system’s status and even propose corrective actions when needed [7]. CNC machine tools are prime candidates for integration with such process monitoring models. The machine tool provides useful process-related data in the form of signals, and the model uses these signals to predict outcomes, such as quality metrics of the produced parts [8] and equipment health [9].

Signals have been widely used as model inputs in the domains of machine health monitoring and predictive maintenance. The typical procedure involves hand-crafted feature design in the time, frequency, or time–frequency domain, followed by feature extraction from the signals and model training [10]. This methodology has been employed in manufacturing for part quality characterization [11,12], chatter identification [13,14], tool wear prediction [15,16], and tool breakage detection [17]. However, manual feature design requires deep expert knowledge, is strictly application specific, and can lead to poor model performance due to inadequate information representation [18], especially for complex domains and multi-sensor setups.

Deep Learning (DL) offers powerful modeling technologies to alleviate these issues, either in combination with traditional techniques or as standalone alternatives [10]. The most prominent types of DL models are Recurrent Neural Networks (RNNs) and Convolutional Neural Networks (CNNs). RNNs had been the standard for processing sequential data before Transformers emerged in 2017 [19]. These networks capture the temporal relationships of the elements in their input and have been successfully applied in tasks such as time-series classification, prediction, and language processing. However, they face difficulties with long sequences due to memory limitations and the vanishing or exploding gradients problem [20]. Long Short-Term Memory networks (LSTMs), a more advanced form of RNNs, offer improvements but they still struggle with processing high-rate signals, such as the ones common in industrial applications. For this reason, they must typically be combined with a feature extraction step [21,22], focusing on learning temporal dependencies between features instead of the elements of the raw input.

CNNs operate by extracting local information from the input arrays in the form of feature maps [20,23]. With each successive convolutional layer, these feature maps detect increasingly complex patterns that underlie the specific characteristics of the input data. Due to this capability, CNNs have contributed significantly to the Artificial Intelligence boom of the 2010s. They find diverse applications, from image recognition [24,25,26] to state representation in sophisticated reinforcement learning frameworks [27,28].

In process monitoring, CNNs offer the potential to avoid the manual feature design and extraction steps in favor of adaptive and arbitrarily rich feature representations, which are integrated in the model itself. Since most CNN architectures were originally designed for two-dimensional (2D) images, transforming signal data into 2D form was a logical approach. In CNC turning, Ibarra-Zarate et al. [29] used 2D CNNs on images of the Mel-Frequency Cepstral Coefficients obtained from acoustic emissions to predict surface roughness. Kuo et al. [30] applied 2D CNNs on fractional order chaos maps of vibration data for chatter detection. Hanchate et al. [31] proposed a framework for predicting average surface roughness in CNC grinding, employing 2D CNNs on time–frequency spectrogram frames of vibration signals. Furthermore, they implemented an explainable AI methodology to identify the most pertinent time–frequency bands influencing the predictions. A similar technique was used by Tran, Liu, and Tran [32] to detect chatter in CNC milling. They applied 2D CNNs on scalograms of the continuous wavelet transform of cutting force signals to classify cutting regions as stable, transitive, or unstable. Kounta et al. [33] focused on chatter prediction via transfer learning. They developed a classifier by fine-tuning pretrained deep 2D CNNs on the normalized Fast Fourier Transform (FFT) images of vibration data. Transfer learning was also employed by Unver and Sener [34] on intrinsic mode functions of cutting force signals. They proposed a combination of 2D CNNs and analytical solutions of the stability boundary for chatter detection in shoulder milling.

CNNs, however, are not restricted to 2D inputs. Signals are one-dimensional (1D) arrays and using 1D convolutions on them removes a preprocessing step while retaining all feature extraction abilities. Additionally, working with 1D data can result in more lightweight models that run faster and require less memory. In the industrial domain, 1D CNNs have been successfully applied to fault detection in rotating machinery, either directly on the raw time-series data [35,36,37,38] or on frequency-domain data, obtained after applying the FFT [39,40].

This approach has recently started gaining traction in manufacturing, predominantly in CNC milling. Zhang et al. [41] employed CNNs with 1D-adapted inception modules and residual blocks for chatter identification based on raw cutting force signals. Lu et al. [42] developed vibration-based 1D CNN models to predict chatter during milling of thin- walled parts. The CNNs are assisted by an attention mechanism that adaptively identifies information-rich frequency bands, while reducing noise interference. Huang and Lee [43] applied 1D CNNs to raw vibration, acoustic emission, and spindle current signals to estimate tool wear and surface roughness development. They trained separate models for each metric and performed an influential sensor selection analysis, which preserves estimation accuracy. Lin et al. [44] also developed 1D CNN regression models for surface roughness and reported the superior performance of CNNs when directly compared with FFT feature extractors.

From the above analysis, it is evident that in the realm of process monitoring there is a recent trend to move away from manual feature extraction methods, with deep learning approaches that employ CNNs showing promising results. Much of the research has focused on chatter detection and tool wear prediction. In terms of part quality, surface roughness has garnered considerable interest while there is a lack of attention to other important quality indicators, such as dimensional accuracy. Regarding implementation, the majority of classification models are binary, for example, detecting stable or unstable cut. Regression models can effectively work only when the machine is actively cutting. To the authors’ best knowledge, all individual models monitor a single metric.

**Figure 1:**
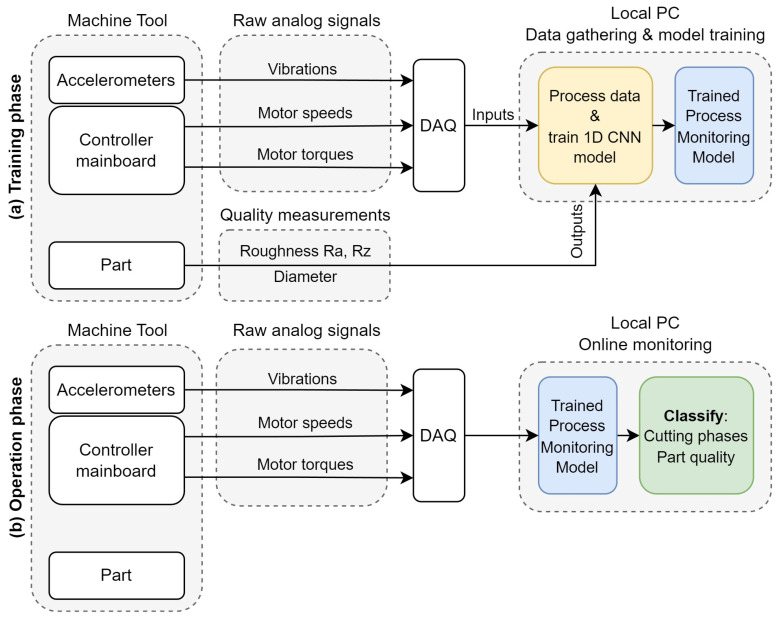
Overview of the proposed methodology: (**a**) Training phase; (**b**) Operation phase.

This study addresses the need for part quality prediction in the context of a process monitoring framework in manufacturing. Recognizing the limitations of traditional feature extraction methods, the proposed methodology employs 1D CNNs on raw time series data from a CNC turning center, including vibrations, motor speeds, and motor torques, see Figure 1. Training data are acquired via a small number of experiments with varying process conditions to produce variability in the monitored metrics. A sliding window technique augments the dataset and allows for continuous monitoring of the entire process. Three quality metrics are selected: arithmetic average roughness (Ra), average peak-to-valley roughness (Rz) [45], and diameter deviation from the nominal value. The classification approach allows for the incorporation of labels not necessarily related to quality assessment. In this case, the model can detect when the machine is not cutting, eliminating the need for external triggers to initiate the inference process. All three metrics, including the non-cutting condition, are monitored by a single model, resulting in an efficient and easily deployable process monitoring framework. Furthermore, several instances of the model are trained for various combinations of design parameters, validating the robustness of the methodology and network architecture under various conditions.

The rest of the paper is organized as follows: Section 2 details the equipment and experimental procedure and presents the experimental data. It also delves into the mapping between signals and quality measurements. Section 3 analyzes the design of the classes, the preparation of data for neural network training, and outlines the network architecture. Section 4 presents and discusses the model’s performance under various conditions, including multiple combinations of design parameters, reduced input from a subset of available signal channels, and alternative definitions of classes. A datastream simulation demonstrates the model’s capability to closely follow a machining process in near-real-time. Section 5 highlights the main conclusions of the study and ends with a discussion on potential future work.

## 2. Experimental Setup

### 2.1. Machine Tool and Instrumentation

**Figure 2:**
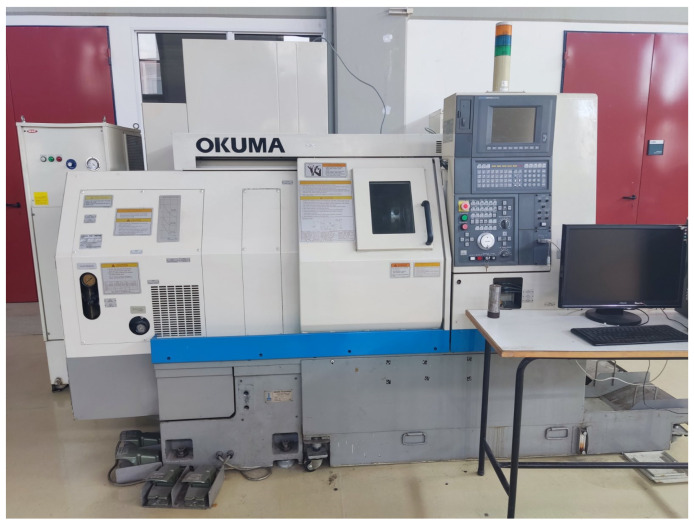
OKUMA LB10ii turning center.

Machining experiments were conducted on an Okuma^TM^ LB10II turning center, equipped with an OSP700L controller, as shown in Figure 2. The machine tool has a maximum turning diameter of 170 mm, a maximum part length of 300 mm, a maximum spindle speed of 10,000 RPM, and 12 turret positions. It has a serial port with DNC capability, which is used to load the G-code. The cutting inserts used are Sandvik Coromant^TM^ CNMG 12 04 08-MR 4305, mounted on a Sandvik Coromant^TM^ PCLNR 2020K 12 tool holder.

**Figure 3:**
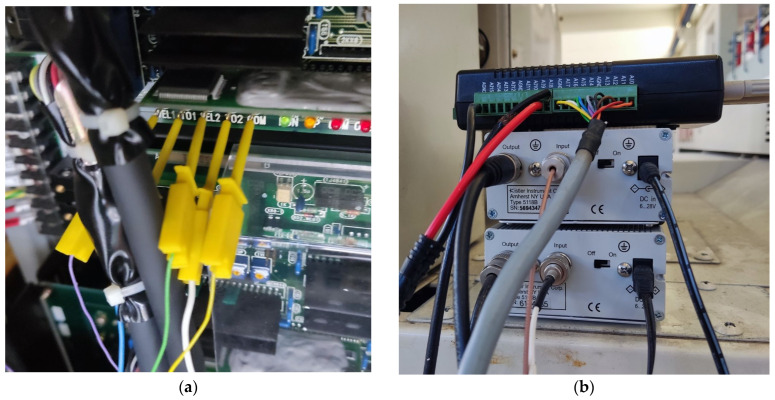
(**a**) Connections to machine controller; (**b**) DAQ and amplifiers.

The machine tool lacks connectivity for providing real-time process data such as spindle rotational speed (RPM), axes linear velocities, and cutting forces. To address this, a direct connection was made to the machine controller mainboard, which provides raw analog signals for motor speeds and torques. The signals for motor speeds correspond to the spindle’s rotation speed and the linear velocities of the X (radial) and Z (longitudinal) axes. Motor torques correlate with cutting force components. Connection to the controller pins was established using hook-type connectors, see Figure 3a. The other ends of the connectors were soldered to a 10-pin port bolted in a slot that was cut in the machine cover. In essence, the machine tool was fitted with a custom-created port, facilitating straightforward physical access to the controller’s raw analog signals for the data acquisition system.

**Figure 4:**
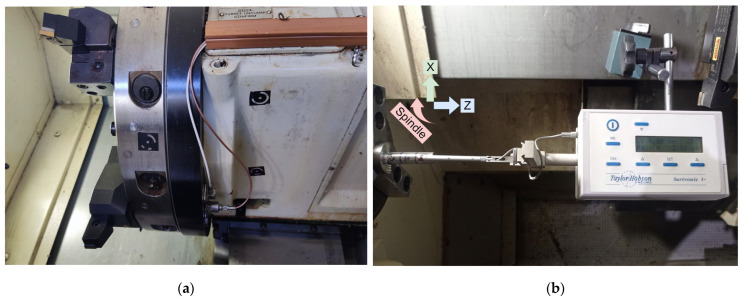
(**a**) Placement of accelerometers behind the machine tool turret; (**b**) Platform for measurement of surface roughness.

For the acquisition of vibration signals, two single-axis accelerometers were used: a KISTLER Type 8640A50 with a ±50 g range and a KISTLER Type 8704B500 with a ±500 g range. Ideally, accelerometers would be mounted directly on the tool holder. However, the presence of a turret, which rotates to change tools, renders this approach unsafe. Additionally, equipping each potential tool required for a given process scenario with dedicated accelerometers is prohibitively costly for a practical application. The proposed solution involves mounting the accelerometers behind the turret using a common magnetic base, see Figure 4a, aligning their axes of measurement with the axes of the turning center (X and Z). The exact placement of the accelerometers is not critical. However, it is crucial that they remain stationary once installed, as moving them would alter the signal characteristics between runs, impacting both experimental results and operational monitoring. This placement results in vibrations traveling through a kinematic chain containing multiple structural elements of the machine before reaching the sensors. Consequently, the signals are weakened, and the readings obtained do not correspond exclusively to vibrations on the expected axis. Instead, the vibrations detected are vectors with components from all axes, indicating a more complex signal composition. The hypothesis behind this sensor placement was that crucial process characteristics are retained in the signal, and neural networks would be capable of discerning them. Successfully training these models should demonstrate their robustness, even when relying on far from ideal input data.

The accelerometers were connected to KISTLER amplifiers (Type 5118B2) using low-noise coaxial cables to ensure high-quality signal transmission. A 10× gain was selected for the second, lower sensitivity accelerometer to bring both sets of readings to the same scale. Accelerometer and motor signals were passed through an ADVANTECH USB DAQ (Type USB-4711A) as shown in Figure 3b to ensure signal synchronization and to facilitate connection to a PC gateway via USB port. The DAQNavi software (version 4.0.9.0) was employed for signal acquisition.

Signals were recorded with a sampling rate of 1 kHz. All types of acquired signals along with the respective DAQ channels are presented in Table 1.

Part quality measurements were carried out manually using relevant instruments after each cut and without removing the part from the spindle. Part diameter was measured with a Tesa^TM^ Micromaster digital CAPA µ micrometer. For surface roughness, a Taylor Hobson^TM^ Surtronic 3+ was employed, mounted on a custom jig with a magnetic base, see Figure 4b. This setup enabled quick and stable mounting and easy dismounting after each set of measurements.

### 2.2. Experimental Procedure

The experimental strategy involves taking longitudinal passes of a specific length on cylindrical workpieces, using various combinations of process parameters, namely depth of cut, feed, and cutting speed, and recording the signals described in Section 2.1. After each pass, the process is halted, and manual quality measurements are performed at specific positions along the workpiece. For surface roughness metrics, Ra and Rz are selected. For dimensional accuracy, the part diameter is measured, which allows for the calculation of diameter deviation (Ddev) from the nominal value, as specified by the G-code.

**Figure 5:**
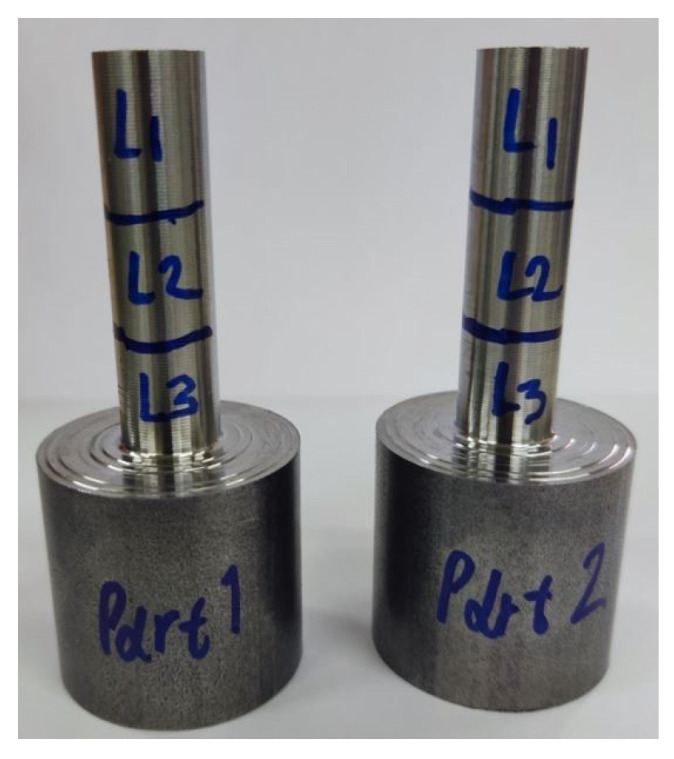
Machined specimens. L1, L2, and L3 denote measurement regions.

The bar stock material is CK45 steel with an initial diameter of 32 mm and a total length of 75 mm. The machined length is set to 45 mm, allowing for the clamping of the workpiece to the chuck and providing some leeway for safety. Quality measurements are taken from three regions, each 15 mm long, denoted as L1, L2, and L3, as shown in Figure 5. These regions accommodate the stroke length of the Taylor Hobson instrument. The part diameter is measured at the midpoint of each region. Each individual measurement is repeated three times at 120-degree angular intervals, and the average of these measurements is taken as the final value. A python script automatically extracts G-code files for each set of process parameters. Constant linear cutting speed is employed (G96 command) and pauses for manual measurements after each pass are specified using the M1 command (optional stop). The cutting fluid utilized is Premiercut GP Semi-Synthetic Cutting Fluid at a 5% dilution, in accordance with the manufacturer’s specifications.

To produce variability in the quality metrics with a limited number of experiments, an orthogonal array is employed with four levels for each of the three factors, as presented in Table 2, resulting in a total of 16 experiments (i.e., passes). The spindle speed is also calculated, due to the G96 command, to ensure that the max rotational spindle speed of the machine tool is not exceeded. Each set of eight consecutive passes, with the specific sequence of depths of cut, reduces the initial diameter of the workpiece by 20 mm. Therefore, starting from a diameter of 32 mm, the part is machined down to a final diameter of 12 mm, at which point a new workpiece is used. For the specified design, only two parts are required. Taking consecutive passes on the same part ensures that different diameters are exposed to a variety of process conditions. This effectively introduces a fourth factor in the experimental design, the slenderness of the part, as expressed by the Length-to-Diameter ratio (L/D). This variation in stiffness broadens the coverage of the process input space, potentially leading to greater variation in the measured quality metrics. Given that the length is constant in this case, the diameter itself is a representation of this slenderness.

### 2.3. Mapping Signals to Quality Measurements

In the proposed model, signals are inputs and quality metrics, which are expressed as labels corresponding to specific ranges of these metrics, are outputs. These data need preprocessing in order to map quality measurements to the appropriate signal segments so that the input–output pairs can ultimately be constructed. To ensure a continuous signal history for each of the two specimens, the first step involves merging the signals from individual passes into a single, continuous signal for each part. This step is required because the process and data acquisition are paused after each pass to allow for manual measurements. The continuous signals contain both cutting and non-cutting regions. The cutting regions, where the tool is engaged with the workpiece, have a direct correspondence to the measured metrics. Non-cutting regions are a result of the free movement of the axes as the machine repositions the tool to follow the process plan indicated by the G-code.

The next step is to identify these cutting regions based on information included in the signals. The most appropriate signal on which to base this segmentation is spindle speed. This is expected to remain constant at various levels for the duration of a pass. Due to the usage of G96 (constant linear cutting speed), the smaller the machined diameter, the higher the spindle speed plateaus, for a given cutting speed. The raw, noisy spindle speed signal is cleaned by applying a moving average with a window of 100 ms. The cleaned signal is then numerically differentiated, and the flat regions, where the gradient is below a threshold of 0.03, are identified. Flat regions shorter than 500 ms are disregarded to exclude irrelevant periods, such as when the spindle is re-engaged after each pause but operates at constant RPM for a brief duration before actual cutting starts. Additionally, any flat regions below a 0.5 V cutoff are also ignored to prevent misidentifying non-cutting regions where the spindle is stopped, and the spindle speed plateaus near zero. The beginning and end of the remaining flat regions denote the timestamps for the pure cutting phases. A custom python script automates the described procedure. The values for the flatness threshold, the minimum segment length, and the amplitude cutoff are given in Table 3. These were determined through experimentation involving trial-end-error and are effective across a wide range of process conditions. From this point forward, the term ‘pass’ will be used to denote each pure cutting region.

**Figure 6:**
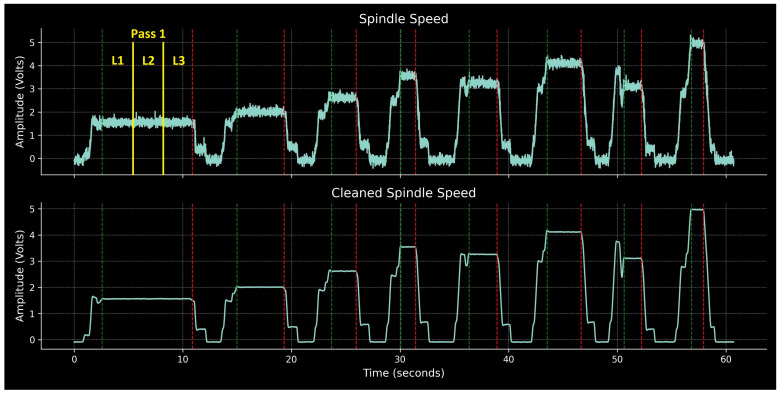
Segmentation of signals based on flat regions of spindle speed for Part 1.

The resulting segmentation into cutting and non-cutting regions is illustrated in Figure 6. This figure displays the spindle speed signals, both raw and cleaned, for the entire history of the first specimen (Part 1). Green dashed lines mark the beginning of each pass, while red dashed lines indicate the end. Outside of these regions, the machine tool is not cutting, i.e., the tool is not engaged with the workpiece. The eight passes corresponding to the first half of the experimental design, which are associated with Part 1, are clearly identifiable. An additional step is then necessary to further segment each pass into the three sub-regions (L1, L2, and L3) at which measurements were taken. This requires knowledge of the measurement strategy, i.e., in our case three equidistant measurements on each pass. This segmentation is indicated by yellow lines in Figure 6, only for the first pass to avoid overcrowding the illustration.

### 2.4. Process Signals

**Figure 7:**
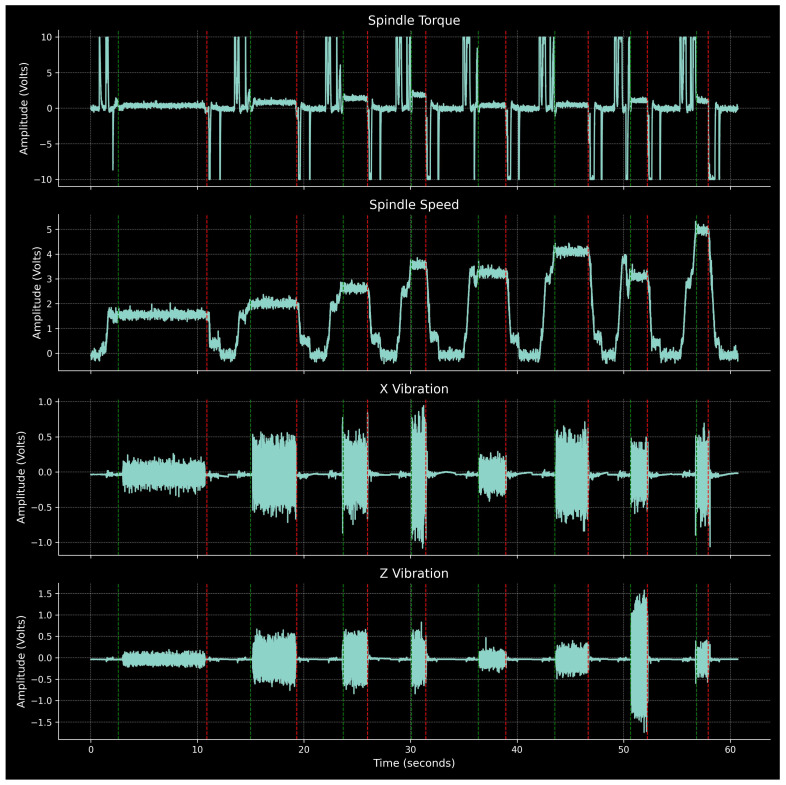
Spindle torque and speed, X and Z vibrations for Part 1.

Figure 7 illustrates the continuous history of Part 1 for the channels corresponding to spindle torque and speed, as well as X and Z axis vibrations. The start and end of individual passes are marked by vertical dashed lines (green for start, red for end). The pure cutting phases are clearly distinguishable in the vibration signals. Their amplitude increases significantly during these periods, from near-zero values otherwise, in alignment with the spindle speed plateaus. This alignment further validates the method of locating cutting phases based on spindle speed. The spindle torque tends to overshoot, either positively or negatively, during spindle start-up, shutdown, or changes in RPM. Notably, within the cutting phases, it stabilizes at a non-zero value, correlating with the tangential cutting force component.

**Figure 8:**
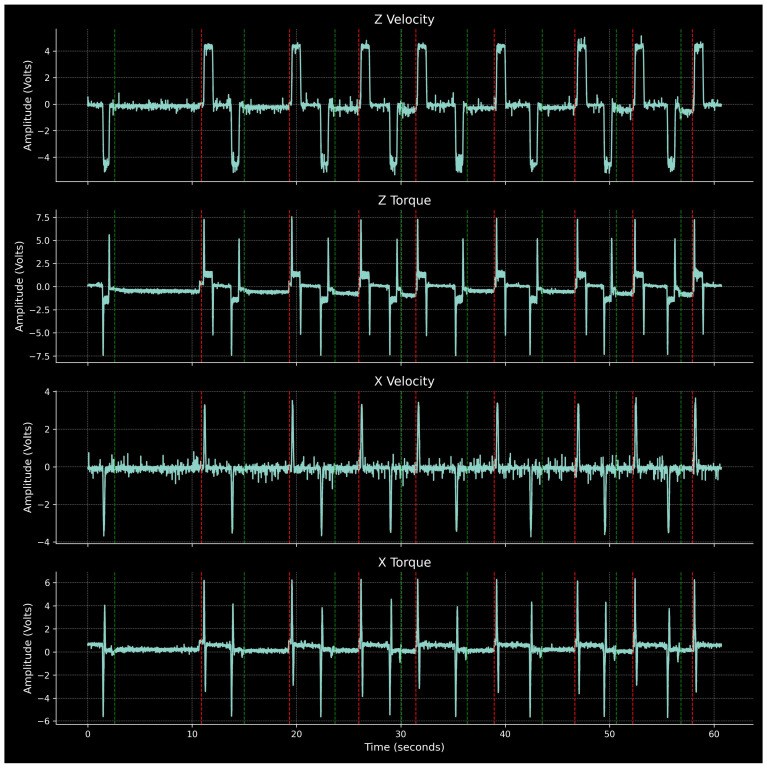
X and Z velocities and torques for Part 1.

The remaining four signal channels are shown in Figure 8. Rapid G00 movements outside of cutting phases result in overshooting that is clearly identified in the X and Z velocity signals. During cutting, the Z velocity takes small negative values corresponding to the feed parameter. This effect is more pronounced during shorter passes, which corresponds to higher feed rates. As expected in purely longitudinal turning, the X velocity remains near zero. Both X and Z torques display inertial readings during axes start and stop phases. The Z torque exhibits correlation with axial force, taking constant negative values during cutting phases. The X torque remains near zero, reflecting the minimal radial force component during cutting.

### 2.5. Quality Measurements

**Figure 9:**
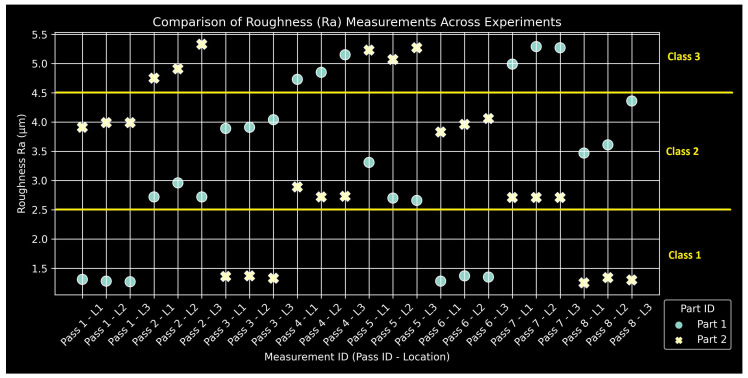
Compilation of Ra measurements with class ranges.

**Figure 10:**
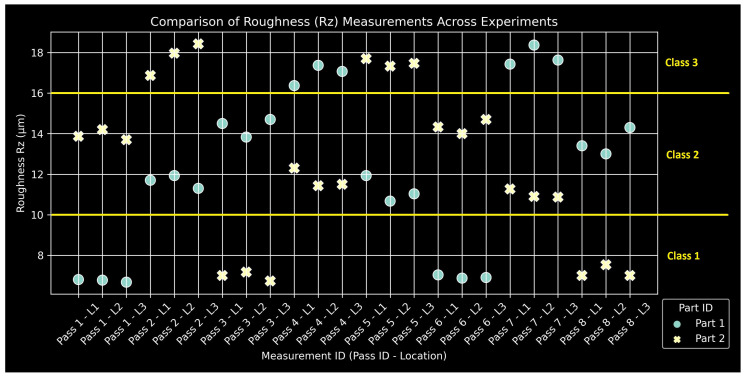
Compilation of Rz measurements with class ranges.

**Figure 11:**
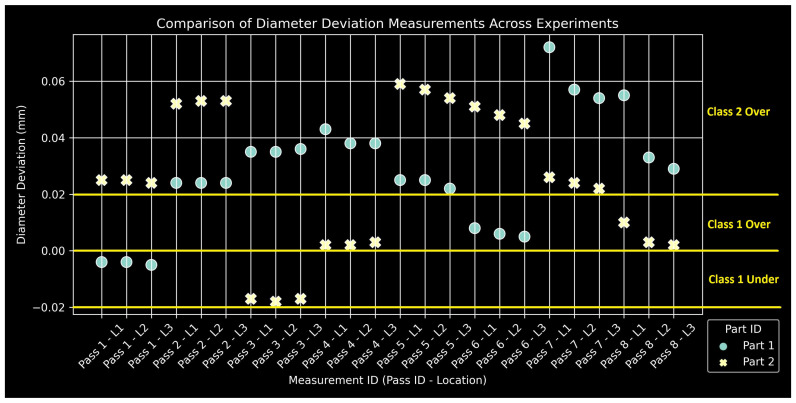
Compilation of Ddev measurements with class ranges.

The manual measurements for all quality metrics of the two specimens are compiled in Figure 9, Figure 10 and Figure 11. Classes and their corresponding ranges, as summarized in Table 4, are indicated with yellow annotations and horizontal lines. The measurement IDs keep track of the pass number and the specific region for each measurement: L1, L2, and L3. The experimental design produced a satisfactory range of values, providing a suitably large output space for neural network training. Measurements tend to form triplets, corresponding to the three length segments of the specimens for a given pass. The data are insufficient to assess the statistical significance of any observed measurement variations along the length of the specimen. At this stage, they are considered within measurement uncertainty for hand-operated instruments.

## 3. Neural Network Design

### 3.1. Data Preparation for Neural Network Training

Convolutional Neural Networks have a fixed input length. Furthermore, the developed model should provide inference during the actual turning process. In order to satisfy both conditions, a sliding window strategy is employed to create the final input–output pairs for neural network training.

Firstly, the quality measurements are categorized into specific classes with corresponding labels, as detailed in Table 4. For the two roughness metrics, three classes of increasing value ranges were created, denoted as Class 1, Class 2, and Class 3. For the diameter deviation, two tight classes at either side of zero deviation were created: Class 1 Under, for diameters smaller than the nominal, and Class 1 Over, for diameters larger than the nominal but within a cutoff that mirrors the negative threshold. A third class, Class 2 Over, encompasses all other greater positive deviations. Lastly, a common label is introduced to each metric to denote non-cutting regions. This additional class is crucial for the neural network’s training, as it includes free axes movements, which are a standard part of any process plan. Consequently, the network can learn to differentiate between cutting phases (where a specific quality label is expected) and Not Cutting phases. Thus, each metric ends up with four classes, leading to an imbalanced multiclass classification problem.

Since there are currently no specifications tied to the quality metrics, the proposed class definitions were based on the following rationale: for Ra and Rz, class ranges are determined with the intent of delineating ‘good’, ‘normal’ and ‘bad’ quality levels. Of course, the range and combinations of process parameters correspond to roughing conditions, meaning that the measured metrics do not dictate the final quality of the part. Thus, within this context, ‘good’ quality may suggest that more aggressive conditions could be feasible. Conversely, ‘bad’ quality might indicate roughness so significant that it could adversely affect a subsequent finishing pass. Although this is speculative and beyond the scope of the study, it does offer a potential perspective. Consequently, Class 1 is narrow in both range and representation. Class 2 is deliberately broader to capture the most common range of measurements. Class 3, equal in representation to Class 1, contains the most extreme results. A similar thought process is behind the diameter deviation class assignment, with the additional consideration that negative values are now possible. This fact naturally leads to two mirrored classes for minor deviations around zero: Class 1 Under and Class 1 Over. These classes could both represent acceptable deviations, though distinctions between over and under sizing may be crucial for specific applications, and the model is capable of making this differentiation. All larger deviations are categorized into Class 2 Over, in order to maintain the same number of classes as for the other two metrics. These class definitions are used in the subsequent results analysis. In Section 4.5, a non-engineered system with equally distributed classes will also be examined.

Having established the class ranges, the actual values of measurements are transformed into the corresponding labels. These labels are then repeated along the signal timeline in accordance with the mapping described in Table 4. Outside of the cutting regions, the label Not Cutting is consistently applied. The final step is to create a sliding window, characterized by its length and a step size that allows for overlap between successive window placements. As the window slides along the signals, it generates input–output pairs for the neural network: the portion of the signals contained within the window serves as the input, and the dominant label for each metric is assigned as the output. Each of these pairs constitutes a single data point. Considering the dominant label as the output handles cases where the window inadvertently spans multiple classes, as is the case, for example, when transitioning from a non-cutting to a cutting region.

This sliding window technique enables the neural network to function as a process monitoring tool. Furthermore, it augments the dataset size. Given the 1 kHz sampling rate, window and step sizes will be expressed in samples, directly corresponding to milliseconds (1 ms per sample). While the window size determines the input size of the neural network, the step size is responsible for the magnitude of this augmentation, as shown in Table 5. For instance, a window size of 500 samples and a step size of 400 samples yield 289 data points from 16 experiments, reducing the step size to 100 samples results in 1153 data points. Both window and step sizes should be tailored to the specific characteristics of each application. In the case presented, a window size of 500 samples and a step size of 100 samples will be the default, with further exploration detailed in Section 4.3. The crucial point to note is that this augmentation allows the model to be effectively trained using data from just two machined parts.

### 3.2. Neural Network Architecture

Typical classification problems have a single output layer. For binary classification, this layer has one output node with a sigmoid activation function, which outputs values from 0 to 1, directly corresponding to the probability of the positive class. In a multiclass problem, the output layer has as many nodes as there are classes, utilizing a single softmax activation. This function converts the combined output of these nodes into a probability distribution, representing the likelihood of each class. For the three metrics of interest—Ra, Rz, and Ddev—three such models could be trained, each monitoring a specific metric. However, this study adopts a more general approach, creating a multi-output, multiclass neural network capable of handling all metrics simultaneously. This requires three separate output layers, each with its own softmax activation. Having a single model predict all three metrics is more challenging, but it offers a versatile framework which covers the cases where either one or multiple metrics are of interest. Additionally, this approach simplifies deployment and enhances inference efficiency, as running a single multi-output model is more time-efficient than running three separate models.

**Figure 12:**
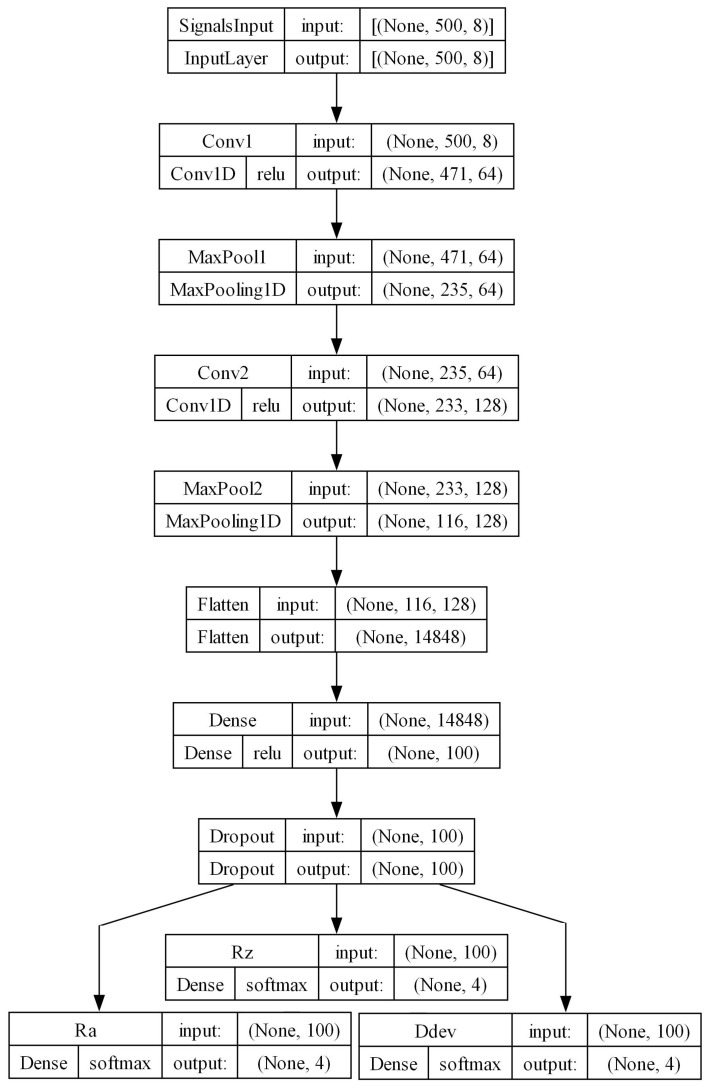
Neural network architecture for window size of 500 samples and 8 signal channels.

The proposed neural network architecture is shown in Figure 12. The shape of the input layer is determined by the window size and the number of signal channels. For example, for a window size of 500 samples and the eight monitored channels (see Table 1), a single input instance to the model is an array of shape 500 × 8. Technically, this forms a tensor of shape N × 500 × 8, where N denotes the batch size. This structure is utilized in neural networks both during training and, when feasible, in inference, to process multiple inputs in parallel.

The input is passed through two convolutional layers with kernel sizes 30 and 3, respectively. The first kernel is relatively large in order to smooth out noise and capture more long-term features in the signals, as suggested by Zhang et al. [35]. The second is of more typical size and it synthesizes first-order features into more complex representations. After each convolution, a max pooling layer reduces the spatial dimensionality and enhances feature robustness by focusing on dominant features. The output from the second max pooling layer is flattened and passed through a dense layer, followed by a dropout layer. This mitigates overfitting by not allowing the network to become excessively dependent on specific nodes. Lastly, three outputs with softmax activations give the probability distribution of classes for each of the metrics of interest. The parameters for all layers are summarized in Table 6. The model was implemented in TensorFlow^TM^ (v2.10.1) using the Keras API.

Depending on the application and the complexity of features in the monitored inputs, more convolutional layers can easily be added, as well as additional dense layers before the outputs. Furthermore, the size of the first, large kernel may be adjusted based on the sampling rate of the signals. For signals sampled in the tens of kilohertz range, it might need to be larger, and conversely smaller for signals sampled at lower rates. Such an investigation is outside the scope of this study, which employs a fixed sampling rate of 1 kHz. The architecture shown represents a lightweight and robust baseline model, which demonstrates strong performance for the task at hand.

### 3.3. Model Training Parameters

Several instances of the model will be trained to thoroughly examine its performance under various scenarios. This section establishes the parameters common to all training instances, unless otherwise specified in the respective sections. These parameters are summarized in Table 7. The detailed results for each examined scenario are presented in Section 4.

## 4. Results and Discussion

### 4.1. K-Fold Cross Validation

Before presenting detailed performance metrics for specific instances of the model, k-fold cross-validation is employed to validate the modeling approach and the network architecture. Instead of using the validation/test split of Table 7, the entire dataset is split into 10 sets. The model is trained 10 times, each time withholding a different set from the training process, which is subsequently used for testing the model’s generalization performance. This strategy mitigates the risk of bias introduced by randomly selecting a favorable test set. Since there is no explicit validation set, early stopping is not employed, and each model is trained for 50 epochs. Models are evaluated by their F1 score, which is the harmonic mean of precision and recall. Results from the cross-validation for all three quality metrics are summarized in Table 8.

The average F1 score across all folds is above 0.97 for all three metrics. The lowest F1 scores for Ra, Rz, and Ddev are 0.94, 0.96, and 0.96, respectively. While some variation in the F1 scores is expected, the consistently high scores across all metrics suggest that the model is robust and performs well on the available data.

### 4.2. Detailed Performance Metrics

**Figure 13:**
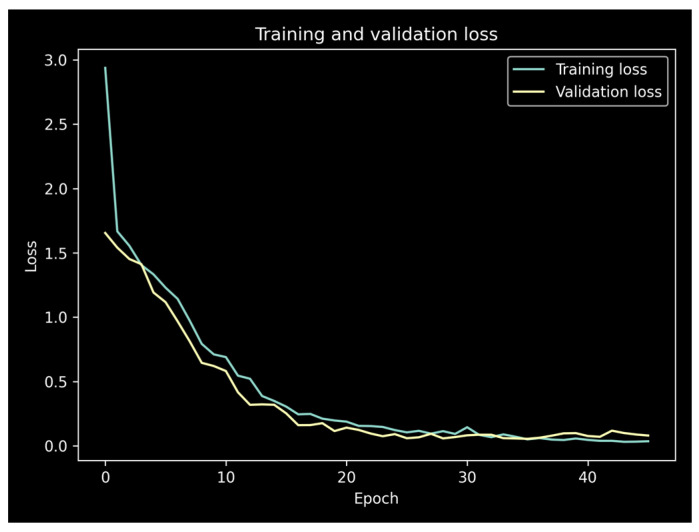
Training and validation loss.

To produce visualizations and detailed metrics for the performance of the model, a specific instance is trained with explicit validation and test sets, according to Table 7. For reproducibility, the split random state for the test set is set to 42. Early stopping is activated with 10 epochs patience for improvement on the validation set and a minimum delta of 0.001. A plot of the training and validation loss is shown in Figure 13. Detailed performance metrics are summarized in Table 9.

**Figure 14:**
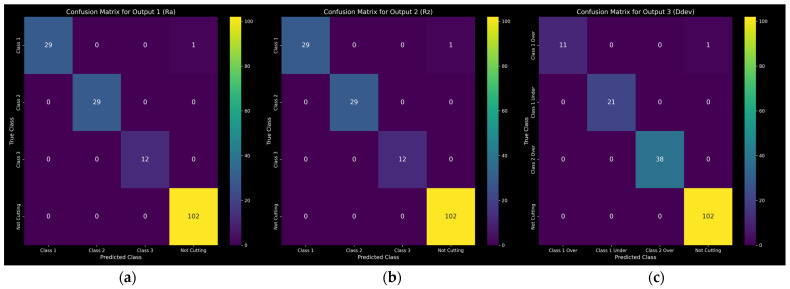
Confusion matrices: (**a**) Ra; (**b**); Rz; (**c**) Ddev.

The model exhibits high precision and recall, within the anticipated margins based on the preceding cross validation. This results in a minimal number of misclassifications, i.e., only 1 out of 173 total classifications for each class, as illustrated in the corresponding confusion matrices shown in Figure 14.

### 4.3. Sliding Window Parameters

The sliding window strategy introduces a form of data augmentation by essentially duplicating sub-segments of signals for successive data points on the signal timeline. This may introduce a positive bias in the testing performance, by having specific sub-segments present in both the training and test sets. On the other hand, increasing the step size reduces the amount of data available for training and decreases the representation of each label in testing. Therefore, a comparison of performance for varying step sizes is not straightforward. To alleviate the diminishing of dataset sizes, validation during training is omitted, and the corresponding data are used for training instead. The models are then trained for 50 epochs for various step sizes. To normalize the differences in support during testing, the F1 weighted average score is utilized, which takes into account the representation of each label in the test set. The window size is kept at 500 samples. Results are given in Table 10.

There is a noticeable trend that as the step size increases, the F1 scores tend to decrease. It is inconclusive whether this is due to the smaller training sets, which may not provide the model with sufficient variability and quantity of data to learn effectively, or to the reduced overlap of data. However, it is noteworthy that for a step size of 500 samples, corresponding to zero overlap of data, the model’s performance could still be considered satisfactory, even with an extremely small training set of less than 200 data points.

To further test the robustness of the model against the sliding window parameters, several instances are trained for varying window sizes. The step size is kept constant at 100 samples, which results in very similar dataset sizes. Results are summarized in Table 11. No noticeable trend emerges, the observed variations being within the expected margin for the inherent randomness in training. Thus, the model exhibits the ability to accommodate various window sizes, depending on the requirements of a given application. Notably, for window and step size equal to 100 samples, which again corresponds to zero overlap of sub-segments but with a sizeable dataset this time, the model’s performance remains extremely high. Therefore, whether overlap exists or not, it does not appear to have a significant impact on the model’s performance. As anticipated, the dataset size is a much more influential factor.

### 4.4. Reduced Input

So far, all eight of the available signal channels were used as inputs to the model. However, depending on the built-in instrumentation of a given machine tool and the availability of external sensors, alternate use cases may have access to a subset of process data. Testing all possible combinations of potential input channels would be impractical, therefore some logical subsets are tested. Additional interesting combinations arising from the initial results are also examined, all summarized in Table 12.

The worst model performance is observed when relying solely on vibrations. This can be attributed, to an extent, to the placement of the accelerometers behind the turret, resulting in a weakened signal with a correspondingly lower signal-to-noise ratio that is also potentially contaminated with natural frequencies of various machine components as explained in Section 2.1. The high performance of torques on the diameter deviation metric can be attributed to their correlation with cutting forces, which are primary factors for part deflection. On the other hand, the high performance of velocities on roughness metrics highlights their strong dependence on the feed parameter. To elaborate further, since the turning is purely longitudinal, the feed in the z-direction should be the most critical velocity. Indeed, using only the Z velocity signal yields very good performance, considering it involves using only one channel, and this performance surpasses that of any other single channel that was tested. Finally, combining torques and Z velocity provides a candidate for the best minimum set of inputs, with performance on par with the full set.

### 4.5. Alternative Definition of Classes

The rationale behind the definition of classes for the quality metrics was discussed in Section 3.1. In this section, the performance of the model is examined for a less ‘engineered’ class system, which simply has a number of equally distributed classes in the observed range of measurements for each metric. This offers the chance to test the model with a different number of classes as well. The results are summarized in Table 13. The ‘+1’ in the number of classes denotes the common class ‘Not Cutting.’ Both the F1 macro and the weighted average are reported since as the number of classes increases, some are left with extremely low support, making the weighted average potentially more descriptive.

**Figure 15:**
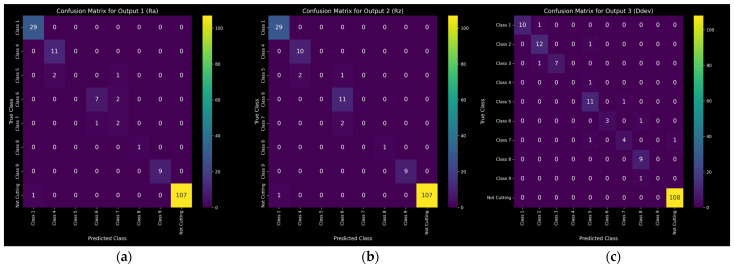
Confusion matrices for 9 + 1 equally distributed classes: (**a**) Ra; (**b**) Rz; (**c**) Ddev.

Both F1 scores are extremely high across all metrics for 2 + 1 and 3 + 1 classes. As the number of classes increases from 5 + 1 and above, the F1 macro scores tend to drop. This is expected, as the size of the dataset is not large enough to accommodate adequate learning examples for so many classes. Furthermore, the support for some classes in the testing set ends up too low, even zero in some cases, leading to ill-defined F1 scores. In cases like these, a single misclassification can dramatically change the macro average. This is reflected in the weighted averages, which maintain higher values. The confusion matrices for the 9 + 1 case are shown in Figure 15. For the two roughness metrics, Classes 2 and 3 have zero instances in the entire dataset, including the training and testing sets, and thus are not included in the confusion matrices. All 10 classes are represented for diameter deviation. Despite the large number of classes for the given dataset size, the performance of the model is deemed very satisfactory.

### 4.6. Datastream Simulation

The proposed model is designed to be lightweight in order to run during the actual turning process. Pure inference times are very low, either on the CPU or GPU of a typical home PC, clocking at under 20 ms. This inference time does not account for data transfer from and to memory, which is typically not an issue when performing batch processing, where data are transferred once, and inference is carried out for the entire batch in the reported times. During real operation however, the situation is different. Batch processing is not an option since data are being generated in real time from the machine, therefore the model needs to process a single data point at a time, which is extremely inefficient for a neural network. The extra overhead in this case is comparable to the inference time, resulting in a total processing time for a data point closer to 50 ms.

**Figure 16:**
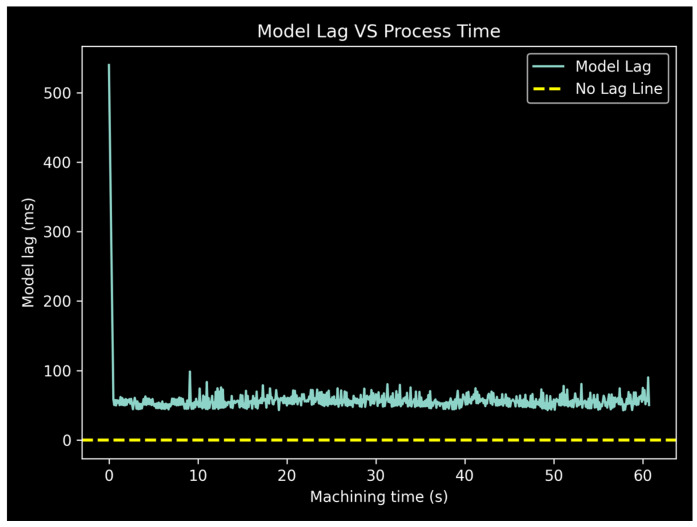
Simulation of model lag VS real process time.

In order to verify the viability of the model in a realistic operation scenario, a datastream simulation is set up. The signal history of the first specimen is used as input. All data points are prepared according to the sliding window strategy, but the model is only allowed to process each of them at specified time intervals, simulating the real data acquisition procedure with a sampling rate of 1 kHz. First, the model has to wait for 500 ms for the first 500 samples window to fill. For each subsequent inference step, the wait time is 100 ms, corresponding to the step size of 100 samples. For each inference step, the exact processing time is calculated and subtracted from the wait time, simulating the continued data acquisition during model inference. The lag of the model versus the real machining time is plotted in Figure 16.

As anticipated, for a total processing time for each data point less than the step size of the sliding window, the model does not drift away from the machining process. After an initial large lag which corresponds to the filling of the first window, the model quickly settles a few tens of milliseconds behind the real process. Random lag spikes may occur, due to arbitrary processes running on the test PC (i5 class CPU, 1060-3GB GPU, 16 GB RAM), however, the model quickly returns to its usual slight delay behind the process. This is presumed to represent an unfavorable scenario where a single-threaded Python program is responsible for performing the datastream simulation, including data transfer, time keeping, and inference. Thus, the model should not drift as long as the sliding window step size is larger than the average processing time of each inference step. Furthermore, there are techniques to further minimize processing times in a real application, such as deployment in optimized hardware environments or using more efficient computing frameworks, which are out of the scope of the present work.

## 5. Conclusions and Future Work

This study presented an innovative application of 1D convolutional neural networks for raw signal classification in CNC process monitoring. The proposed methodology eschews manually engineered feature extraction, typically employed in signal processing, in favor of temporal pattern identification by the neural network itself. Convolutional layers identify and synthesize pertinent, process-related information contained in multiple signal channels acquired from the machine tool, in order to classify the machining process with respect to its resulting quality. It was shown that with an extremely small number of experiments, in full compliance to industry expectations, and a sliding window strategy, a multi-output, multiclass model can be successfully trained to monitor several quality metrics at once. The performance of the model was investigated under various combinations of design parameters and constraints, and it proved to be a robust base for real-time monitoring and quality control in CNC machining. Furthermore, this approach provides a versatile alternative for the classification of high-rate signals across various industrial domains where such analysis is relevant.

To build upon the presented work, several potential avenues are available. Conducting more experiments will result in greater coverage of the input space of process conditions, as well as the output space of quality metrics. Moreover, additional experiments will increase the raw size of the dataset, which is generally beneficial to model training. Including finishing conditions in the experimental design will offer a complete view of a typical real machining application. In this context, it will be interesting to investigate the potential adverse effects of an aggressive roughing pass on a subsequent finishing pass. This could be valuable in the context of a process optimization framework, which can suggest efficient process planning while ensuring final quality. Further considerations relating to tool wear and the sustainability of the process can also be explored in this light.

Finally, testing the model during real machining operations presents its own set of challenges, associated with model deployment, establishing communication protocols, and ensuring seamless integration into the existing workflow. Addressing these challenges is crucial for the successful application of the model in a live industrial setting.

## Figures and Tables

**Table 1:** DAQ setup with acquired signals and respective axes.

**DAQ channel**	2	3	4	5	6	7	8	9
**Axis**	Spindle	Spindle	Z	Z	X	X	X	Z
**Signal (V)**	Torque	Speed	Velocity	Torque	Velocity	Torque	Vibration	Vibration
**Source**	Machine controller						Accelerometers	

**Table 2:** Experimental design.

Experiment No	Workpiece Id	Depth of Cut (mm)	Feed (mm/rev)	Cutting Speed (m/min)	Final Diameter (mm)	Spindle Speed (RPM)
1	Part 1	0.5	0.2	160	31	1642.89
2		1	0.3	190	29	2085.48
3		1.5	0.4	220	26	2693.39
4		2	0.5	250	22	3617.16
5		0.5	0.3	220	21	3334.67
6		1	0.2	250	19	4188.29
7		1.5	0.5	160	16	3183.1
8		2	0.4	190	12	5039.91
9	Part 2	0.5	0.4	250	31	2567.02
10		1	0.5	220	29	2414.76
11		1.5	0.2	190	26	2326.11
12		2	0.3	160	22	2314.98
13		0.5	0.5	190	21	2879.95
14		1	0.4	160	19	2680.5
15		1.5	0.3	250	16	4973.59
16		2	0.2	220	12	5835.68

**Table 3:** Parameters for identifying cutting regions.

Flatness Threshold	Minimum Segment Length	Amplitude Cutoff
0.03	500 ms	0.5 V

**Table 4:** Labels for all metrics with corresponding ranges.

Metric	Label	Range (μm)
Roughness Ra	Class 1	[0, 2.5)
	Class 2	[2.5, 4.5)
	Class 3	[4.5, 10]
Roughness Rz	Class 1	[0, 10)
	Class 2	[10, 16)
	Class 3	[16, 20]
Diameter deviation Ddev	Class 1 Under	[−20, 0)
	Class 1 Over	[0, 20)
	Class 2 Over	[20, 80]
All metrics	Not Cutting	N/A

**Table 5:** Size of dataset for 500 samples window and various step sizes.

Step Size (Samples)	Number of Data Points
100	1153
250	461
400	289

**Table 6:** Neural network layer parameters.

Layer	Parameters
Conv1	kernel 30, filters 64, stride 1
MaxPool1	kernel 2, stride 1
Conv2	kernel 3, filters 128, stride 1
MaxPool2	kernel 2, stride 1
Flatten	-
Dense	size 100
Dropout	rate 0.2
Output X3	3 × size 4
Total trainable parameters: ~1.5 M	

**Table 7:** Default model training parameters.

Parameter	Value
Window size	500 samples
Step size	100 samples
Optimizer	Adam
Learning rate	0.0005
Loss	Categorical cross-entropy
Batch size	128
Max epochs	100
Validation split	0.15 of training
Patience	10 epochs
Min delta	0.001
Test split	0.15 of total
Split random state	42

**Table 8:** *k*-fold cross validation results (*k* = 10).

Metric	F1 Macro Average										
	Fold 1	Fold 2	Fold 3	Fold 4	Fold 5	Fold 6	Fold 7	Fold 8	Fold 9	Fold 10	Average
Ra	0.94	0.97	0.98	1.00	0.97	0.98	0.99	0.95	1.00	0.98	97.76
Rz	0.98	0.97	0.97	1.00	0.96	0.98	1.00	0.97	1.00	0.99	98.34
Ddev	0.97	0.97	1.00	0.96	0.98	1.00	0.99	0.97	1.00	1.00	98.46

**Table 9:** Model performance metrics.

Metric	Label	Precision	Recall	F1-Score	Support
Ra	Class 1	1.00	0.97	0.98	30
	Class 2	1.00	1.00	1.00	29
	Class 3	1.00	1.00	1.00	12
	Not Cutting	0.99	1.00	1.00	102
Rz	Class 1	1.00	0.97	0.98	30
	Class 2	1.00	1.00	1.00	29
	Class 3	1.00	1.00	1.00	12
	Not Cutting	0.99	1.00	1.00	102
Ddev	Class 1 Under	1.00	1.00	1.00	21
	Class 1 Over	1.00	0.92	0.96	12
	Class 2 Over	1.00	1.00	1.00	38
	Not Cutting	0.99	1.00	1.00	102

**Table 10:** Model performance for varying step size and window size of 500 samples.

Step Size	F1 Weighted Average			Data Points (Train/Test)
	Ra	Rz	Ddev	
200	0.99	1.00	0.99	490/87
300	0.97	0.97	0.95	327/58
400	1.00	0.98	0.92	245/44
500	0.91	0.91	0.85	196/35

**Table 11:** Model performance for varying window size and step size of 100 samples.

Window Size	F1 Macro Average			Data Points (Train/Test)
	Ra	Rz	Ddev	
100	0.98	0.98	0.98	986/175
200	0.98	0.98	0.98	985/174
400	0.97	0.98	0.98	981/174
600	0.99	1.00	0.99	978/173
800	1.00	1.00	0.99	974/173
1000	0.97	0.97	0.99	971/172

**Table 12:** Model performance for reduced input.

Subset of Channels	F1 Macro Average		
	Ra	Rz	Ddev
Only vibrations	0.80	0.76	0.71
Only torques	0.94	0.94	0.96
Only velocities	0.99	0.99	0.93
Only Z velocity	0.89	0.90	0.79
Z velocity and all torques	0.99	0.99	0.98

**Table 13:** Model performance for equally distributed classes.

Number of Equally Distributed Classes	F1 Macro Average			F1 Weighted Average		
	Ra	Rz	Ddev	Ra	Rz	Ddev
2 + 1	0.97	0.99	0.98	0.98	0.99	0.99
3 + 1	0.91	0.98	0.97	0.95	0.99	0.98
5 + 1	0.79	0.89	0.98	0.94	0.96	0.99
7 + 1	0.74	0.69	0.64	0.97	0.94	0.91
9 + 1	0.78	0.72	0.71	0.95	0.95	0.94

## Data Availability

This study is part of an ongoing EU funded research project and related data cannot be made public at this time.
